# Social representations of the health care of the Mbyá-Guarani indigenous population by health workers^1^

**DOI:** 10.1590/1518-8345.1505.2846

**Published:** 2017-02-06

**Authors:** Mirian Benites Falkenberg, Helena Eri Shimizu, Ximena Pamela Díaz Bermudez

**Affiliations:** 2 MSc, Researcher, Secretaria Executiva do Conselho Nacional de Saúde, Ministério da Saúde, Brasília, DF, Brazil.; 3 PhD, Associate Professor, Faculdade de Ciências da Saúde, Universidade de Brasília, Brasília, DF, Brazil.; 4 PhD, Adjunct Professor, Departamento de Saúde Coletiva, Universidade de Brasília, Brasília, DF, Brazil.

**Keywords:** Health of Indigenous Peoples, Culturally Competent Care, Medicine, Traditional

## Abstract

**Objective:**

to analyze the social representations of health care of the Mbyá-Guarani ethnic group by multidisciplinary teams from the Special Indigenous Health District in the south coast of Rio Grande do Sul state (Distrito Sanitário Especial Indígena Litoral Sul do Rio Grande do Sul), Brazil.

**Method:**

a qualitative method based on the theory of social representations was used. Data were collected via semi-structured interviews with 20 health workers and by participant observation. The interviews were analyzed with ALCESTE software, which conducts a lexical content analysis using quantitative techniques for the treatment of textual data.

**Results:**

there were disagreements in the health care concepts and practices between traditional medicine and biomedicine; however, some progress has been achieved in the area of intermedicality. The ethnic boundaries established between health workers and indigenous peoples based on their representations of culture and family, together with the lack of infrastructure and organization of health actions, are perceived as factors that hinder health care in an intercultural context.

**Conclusion:**

a new basis for the process of indigenous health care needs to be established by understanding the needs identified and by agreement among individuals, groups, and health professionals via intercultural exchange.

## Introduction

Indigenous health care in Brazil is conducted within the framework of the indigenous health care subsystem created by Law No. 9,836/1999 in partnership with the Unified Health System (Sistema Único de Saúde - SUS). The subsystem is structured to include 34 Special Indigenous Health Districts (SIHDs). The service organization model on which the system is based includes technical, administrative, and managerial activities, along with social control, and has faced many challenges in the provision of health care to the indigenous population^1^.

Indigenous health care at the primary care level is practiced by members of indigenous health multidisciplinary teams (IHMTs). These teams consist of non-indigenous health professionals, physicians, nurses, dentists, dietitians, and nursing technicians, among others. The teams also include workers known as Indigenous Health Agents (IHAs) and Indigenous Sanitation Agents (ISAs) who are selected or nominated by their communities and trained to work to support health activities. One of the main tasks of these workers is the translation and interpretation of traditional language and knowledge among non-indigenous professionals. IHAs have been the main link between scientific knowledge and the popular knowledge of indigenous communities.

Indigenous health care for the different ethnic groups that compose the indigenous health care subsystem has been characterized by therapeutic plurality, which is the simultaneous use of several health practices, and this is supported by the health professionals^2^. Indigenous people involved in health care services have several designations, including pajes, healers, shamans, chanters, kuiãs, and karaís. These leaders often have political and religious duties. The health care they provide involves various practices, including the use of medicinal plants and various healing rituals that reflect the health concepts associated with complex systems that are configured in the polysemic concept known as traditional indigenous medicine^3^. Since 2002, the World Health Organization (WHO) has encouraged countries to incorporate traditional medicine (TM), particularly in primary care, to increase the access of health agents to populations with cultural differences^3^ ^-^ ^4^. TM involves several social care systems within the field of medicine; in the broad sense, these health practices are established holistically as socially coordinated responses to human diseases^3^ ^,^ ^5^. For these reasons, the comprehensiveness of care of indigenous populations involves a dimension of interculturalism that brings with it the idea of cultural diversity and the relationships between different cultures or, more specifically, between individuals belonging to different cultures^6^.

From a critical perspective^6^, interculturality needs to be understood as a constant movement in search of social, economic, political, and ethnic relationships that are fair, respectful, ethical, and, above all, human. This complex challenge must be viewed as an ongoing process with the purpose of improving relationships between individuals, areas of knowledge, and practices that are culturally different, especially among ethnic groups that are historically subordinate, including indigenous and black groups.

Some legal instruments are available to ensure the comprehensive health care of indigenous peoples considering their social, cultural, geographical, historical, and political diversity. The National Policy for Health Care of Indigenous Peoples (NPHCIP), which was approved by the Ministry of Health’s Ordinance No. 254 dated January 31, 2002, advocates recognition of the effectiveness of traditional medicine and the right of indigenous peoples to their culture. Among its guidelines, the NPHCIP refers to the preparation of human resources to work in intercultural contexts and the cooperation of traditional indigenous systems to ensure the access of indigenous peoples to comprehensive care^1^.

Despite the normative advances of the NPHCIP, in practice it has not yet been possible to consolidate the strategies that value the diversity of opinions and health care systems by addressing ethnic and cultural issues^7^. Above all, it is recognized that the implementation of the principles of differentiated care, which are clearly defined in official documents, requires that multidisciplinary teams understand the lifestyle of indigenous peoples, their social organization, representations of the health-disease process, and cultural specificities, among other factors^8^. Furthermore, it is known that health care services still ignore social involvement, thereby limiting the opportunity for dialogue between Western medical practice and traditional medicine; the latter could contribute to the development of a local health system adequate to the reality of indigenous peoples^8^ ^-^ ^9^.

The unpreparedness of managers who establish these policies and health professionals who carry them out to address indigenous issues in the perspective of interculturalism is evident^10^ ^-^ ^12^. One reason for this is that the university model, which is intended to train professionals in the biomedical and medicalized model, is considered hegemonic, superior, and irreplaceable^13^ ^-^ ^14^.

This study addresses various concepts of health, disease, and indigenous health care by evaluating how health workers who are trained in biomedicine, who have disease as a guiding principle of their practices, and who are aided by indigenous workers represent health care at the intersection between two distinct medical systems, biomedicine and the *Mbyá*-Guarani traditional indigenous medicine.

Moreover, this study examines the hypothesis that knowledge of the social representations of care among these workers could improve reflection on the work of indigenous health teams, with a view to proposing strategies that would enhance their potential and help overcome their limitations.

This study aimed to analyze the social representations of health care provided to the *Mbyá*-Guarani ethnic group by multidisciplinary teams from the SIHDs in the south coast of Rio Grande do Sul state, Brazil to assess the potential and limitations of indigenous health care practices.

## Methodology

This qualitative study used the framework of the Social Representation Theory (SRT) created by Serge Moscovici. Social representations consist of common-sense knowledge that guides individual and collective actions^15^ ^-^ ^16^. They are always a result of interactions and information exchange and assume specific shapes and configurations as a result of the specific balance between processes of social influence. Social representations are intended to make something unfamiliar into something familiar and are characterized by a constructive process of anchoring and objectification^15^. Anchoring corresponds to the incorporation of new elements of an object into a system of categories that are familiar and functional to individuals, and objectification aims to make concrete what is abstract, i.e., to transform a concept into an image of something, removing it from its scientific conceptual framework^15^.

 This study focused on the analysis of the contents of social representations brought by health workers and attempted to identify, whenever possible, the anchoring and objectification processes that are considered fundamental in the formation of social representations.

Data were collected in the state of Rio Grande do Sul, which has a population of 2,321 indigenous persons of the Guarani, *Mbyá*-Guarani, and Kaingang ethnic groups. For this study, only the population whose individuals self-declared as *Mbyá*-Guarani was evaluated; this population consisted of 1,015 indigenous people. Participant selection was intentional and theoretical. The study participants included 20 workers who made up 3 IHMTs located in 3 stations, as follows: Barra do Ribeiro station: 1 dentist, 1 nurse, 2 nursing technicians, 1 IHA, and 1 ISA, totaling 6 workers; Viamão station: 1 dentist, 1 nurse, 2 nursing technicians, 3 IHAs, and 2 ISAs, totaling 9 workers; and Osório station: 1 dentist, 1 nurse, 2 nursing technicians, and 1 IHA, totaling 5 workers. Data were collected between January and March of 2014.

Semi-structured interviews were conducted using the following questions: 1. How do you feel working with the indigenous population of the *Mbyá*-Guarani ethnic group? 2. Tell about your professional experience. 3. How do you describe the *Mbyá*-Guarani people? 4. In your opinion, what are the health needs of this population? 5. In your opinion, what indigenous health care would promote the articulation of traditional indigenous systems as recommended by the NPHCIP? 6. Do you know and can you describe some TM practices of the *Mbyá*-Guarani population? 7. In your daily work, have you experienced any difficulty in accomplishing the articulation proposed in the policy? 8. What is easy and what is difficult in your daily work with this indigenous population?

The interviews were conducted during the working hours of health workers in the villages visited, attempting not to interfere with the work routines of the workers. Upon arriving in the villages, the cacique (tribe leader) was immediately contacted to explain the presence of the researcher and the research objectives and to request written permission to conduct the interviews and to observe the participants. The sample size was based on the saturation of the statements contained in the interviews.

Participant observation, which was used as a complementary strategy to the interviews, was essential because it helped support the understanding of health care practices considering the social representations identified. Daily events in the villages, including eating habits, leisure, and adult and child care, were observed and recorded in a field diary with a focus on the impressions of the researcher on intercultural contexts, the people contacted informally, and feelings about and interpretations of what was seen, heard, and spoken, as well as acceptance of the interviews by the group.

The interviews were recorded and transcribed by the researcher, and ALCESTE software (*Analyse Lexicale par Contexte d’um Ensemble de Segments de Texte*) version 4.10 was used as a support for data content analysis. This software was developed by Max Reinert in 1990 in France and is used to perform statistical analysis of written text data. Each of the twenty interviews constituted an initial context unit (ICU). The set of ICUs constituted the study corpus.

The software was used to perform 4 analyses of the input data (stages A, B, C, and D). The first 3 stages (A, B, and C) involved 3 operations, and the fourth stage (D) involved 5 operations, as follows: *Stage A* - reading of the text and calculation of dictionaries: stage A1 - reformatting and division of the text into similar-sized segments; stage A2 - search of the vocabulary and reduction of words considering their roots; and stage A3 - creation of the dictionary containing reduced word forms; *Stage B* - calculation of data matrices and classification of elementary context units (ECUs): stage B1 - selection of ECUs to be considered and calculation of the matrix:reduced word forms X ECUs; stage B2 - calculation of data matrices for descending hierarchical classification (DHC); and stage B3 - DHC; *Stage C* - description of ECU categories: stage C1 - definition of the chosen categories; stage C2 - description of the categories; and stage C3 - correspondence factor analysis (CFA); *Stage D* - additional calculations: stage D1 - selection of the most characteristic ECUs of each category; stage D2 - search for repeated segments in each category; stage D3 - ascending hierarchical classification (AHC); stage D4 - selection of the most characteristic words in each category and creation of a frequency-of-occurrence index; and stage D5 - export to other sub-corpus ECU software in each category.

ECUs are text segments containing an average of three lines that are created by the software depending on the size of the corpus. These ECUs correspond to the most calibrated sentences according to the text size and score. As explained in the previous paragraph, the ECUs were classified during Stage B of the process, and categories that represented themes extracted from the text were then obtained from this classification. The vocabulary in each category is similar, but it differs from the ECUs in other categories. The chi-square test is used to evaluate the association of the reduced forms and the ECUs with the categories. At this stage, the software offers the researcher one of its most useful features: it organizes the data provided as a DHC, which allows lexicographical analysis of the textual material and the introduction of contexts characterized by their vocabularies and by the text segments that share this vocabulary. This first classification by the software divides the partitions of the corpus into lexical categories and reveals their disagreements using dendrograms. Dendrograms graphically express the categories that reflect the themes extracted from the text; these themes can be described mainly by their characteristic vocabularies. Studies of social representation have used this software because it allows the user to become familiar with the ideas and meanings shared by a particular social group, thus guiding the qualitative analysis of the meanings produced within a given context^12^.

This study was approved by the Research Ethics Committee of the School of Health Sciences of the University of Brasilia (Comitê de Ética em Pesquisa da Faculdade de Ciências da Saúde da Universidade de Brasília - CEP/FS-UNB) under Opinion No. 313,588. Because this study involved indigenous populations, it was also evaluated by the National Research Ethics Committee (Comissão Nacional de Ética em Pesquisa-CONEP) of the Ministry of Health (MH) and was approved under Opinion No. 518,681. All study participants were encoded to preserve anonymity.

## Results

**Figure 1:**
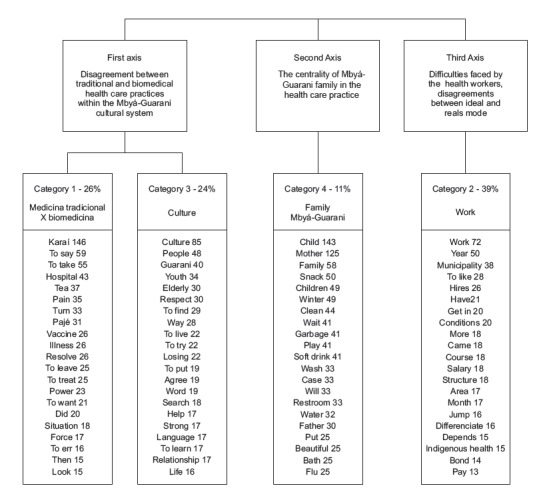
Social representations of health care for the Mbyá-Guaranipeople

The analysis of the corpus led to the following general results: 20 UCIs, 4 stable categories, 50,467 words analyzed, and 1,444 ECUs classified; 63% of the UCEs were classified. The contents of social representations of health care are shown in DHC dendrograms (Figure 1).

The first axis, which includes categories 1 and 3, revealed contents related to the disagreement between the care practices of traditional medicine and biomedicine inserted in the cultural universe of the *Mbyá*-Guarani people. The second axis, defined by category 4, revealed that health care is focused on the families of the *Mbyá*-Guarani people. The third axis, formed by category 2, indicated how the work organization and the lack of infrastructure hinder cross-cultural care.

The first axis, category 1, indicated the predominant concept of traditional practices in the daily life of the tribe members, which generates the need for ongoing negotiation between health workers and the spiritual leaders (*karaí*) of each community to define health care practices. *Sometimes you speak to those who are sick, and they say No! But it is no use to give your remedy to them because their spirit is not here. Their spirit left the body. While the spirit does not come back here, the pajé, the karai that they invoke does not bring the spirit back, there is no point in providing medicine because they will not be healthy!* (TNI 10).

Therefore, traditional medicine is represented by health workers and represents a distinct world that is difficult to understand from the perspective of biomedicine. *And it is complicated because these worlds are different but living together. It is a challenge!* (TI 8).

Category 3 revealed contents related to culture, with an emphasis on the way of being of the Guarani people, which involves their organic interaction with nature and the discrepancy between the maintenance of the cultural system advocated by the leaders, elders, and women in the community and the inevitable changes introduced to the way of life of the *Mbyá*-Guarani people by interethnic contact.

It is of note that the way of being of the Guarani people generates both admiration and distress among health workers because they advocate the maintenance of the cultural system but also realize that the changes resulting from interethnic contact, brought from outside, are aggressive. *At lunch, if you manage to watch you’ll see. They are eating snacks and drinking soda. In every meal, every meal! Then I say: you have to brush your teeth, and I distribute toothbrushes and fluoride, but that is the least I can do! Because their eating habits are not helping!* (TNI 20).

The second axis, consisting of category 4, revealed that family is the focus of health care. That is, care practices occur and are justified in the family, for the family, and by the family of the *Mbyá*-Guarani people. However, among health workers, the concept of family is unique because it involves larger social groups, and health care is referred to as “community work” in which all members work for a common goal.

Category 4 included content related to the poor living conditions of the *Mbyá*-Guarani people, who, in some of the visited villages, did not have access to potable water, sanitation, or electricity. Lack of these items is a determining factor for the emergence of diseases and disorders that affect the Mbyá people, particularly the children.

The third axis, consisting of category 2, explored the lack of infrastructure and health work organization, indicating the difficulties that health workers face, including the casualization of employment contracts, low pay versus the dedication required by this type of work, poor infrastructure of community services (transport, facilities, and supplies) and lack of training projects. The latter primarily impacts new employees because those employed longer acquire practical experience that helps them to be stable. *And it’s something that, as a professional, I would like that so many things were accomplished and became a reality to the indigenous people. And would very much appreciate if these decisions were made by the top end because we are the lower end. And that the top end would follow, and could see this way* (TNI 17).

The contents of the statements above indicate the significant inconsistencies between the ideal service organization models established by the creation of the subsystem and the implementation of NPHCIP and the actual models adopted in everyday work.

## Discussion

Social representations of care among health workers of IHMTs strongly correlated with the traditional medicine of the *Mbyá*-Guarani culture, considering their world, which is spatially well-defined. The *Mbyá* world promotes a different way of being, a different medicine, and a unique form of interpretation of life, death, health, and care. It is referred to as the world of the other, the world apart, their world; therefore, this world should be the focus of health care interventions.

In this perspective, culture is understood as relationships that are based on social experience in which subjectivities such as affection and emotions are manifested and interpreted; this is also the case with the cognitive dimension, which uses socially provided categories. On the other hand, the culture, in this perspective, refers to the metaphor of the Geertzian text, which is not only socially constructed but gains meaning in social life^16^ ^-^ ^17^. Social experience develops as it is recognized, shared, and confirmed by others in their acts of articulation and communication. Therefore, it has an intersubjective and social nature^16^. As a human experience, cultures participate in the construction of the world, bringing an element of transformation.

All subsystems that circulate and intertwine in a complex cultural system, including traditional medical systems, are related to a world view, as observed in this study, and to a corresponding ethos of its people^17^. In this sense, the world view is a cognitive and existential attribute that allows certain people to develop a representation of a simple reality, including its concept of nature, self, and society. The ethos is a moral and aesthetic attribute that reflects the nature and quality of life of a people, and its organization is the underlying attitude towards itself and its world reflected by life^17^.

Another objectification identified in the formation of social representations of traditional medicine was religion. Geertz^17^ states that religion combines ethos and the world view of a people, providing to the set of social values what perhaps they most need to be coercive: a sense of objectivity. Language and religion, which are the unifying elements of the *Mbyá* people, contain information and explanations of the ancient methods of promotion, protection, and recovery of health^7^.

The anchoring of health care in the culture, objectified as a world, indicates the presence of ethnic boundaries that generate constant tensions between concepts and practices in the intercultural contexts studied^18^. This social representation was classified in the scope of intersubjectivity and was developed by verbal communication and cooperation between health workers and indigenous people. Therefore, traditional care and biomedical care practices are represented in different ways because they belong to different worlds, the *Mbyá* world or the world of *Juruá* (white men). However, it is clear that this form of individualized classification increases the distance between the two worlds when approximation between the two worlds is expected in health care practices in the context of interculturalism.

In the world of the *Mbyá*, health workers and their care practices are foreign elements endowed with the power of biomedicine, i.e., knowledge with more value, to the detriment of indigenous knowledge, which is often considered to consist of ineffective beliefs^18^. This contrast causes tension among health workers because they feel guilty for interfering in this world but also gratified for helping those in need. The attempt to find a balance is ongoing, with most workers striving not to interfere too much but obliged to fulfill their duties as biomedicine workers.

In this perspective, each of these two medical systems, traditional medicine and biomedicine, is part of a specific cultural dimension that coexists within the Mbyá world, leading to a relationship that triggers the formation and sharing of social representations of care among IHMT workers and to a consensual, confined, and redefined world of health care^15^.

We also found that the coexistence of these two medical systems has prompted the health teams to work cooperatively over time to seek solutions to practical daily issues. This cooperation, known as intermedicality, aims to facilitate the interaction between knowledge based on biomedicine and non-medical knowledge^2^. These cultural competencies are required by workers to minimize the conflicts between different cultures^13^.

The anchoring of health care with a focus on the family indicated the central role of the care recipients, the *Mbyá*-Guarani people. Therefore, the IHMT members have focused health care in the family. However, family care is different because the family represents the collective. The social organization of the Guarani people is based on large families in which children grow up among many people and do not focus their emotions and expectations for reward and punishment on a few individuals or on specific individuals^19^.

For health workers, the family is represented by larger groups of individuals and by whole communities, and this concept brings feelings of satisfaction and accomplishment when community work improves collective health regarding water sanitation, community-based cleaning activities, and garbage collection. On the other hand, it creates distress by representing the families, especially children, as victims of care strategies that are considered inadequate by the community, particularly concerning hygiene and nutrition.

Contradictions are observed in the way the *Mbyá* family is represented, sometimes with admiration, sometimes with discrimination, especially when health workers evaluate the care of children by the parents with respect to eating habits and hygiene. These representations may be partially responsible for the difficulties workers encounter in developing educational activities for the promotion of health. Moreover, these representations may camouflage the perception of a broader social, economic, and political context of structural violence related to poverty and ethnic discrimination, which creates poor living conditions as well as limited access to land, drinking water, electricity, and health^19^. These problems are due to increasing aggression from interethnic contact, designated interethnic friction; such aggression has resulted in the elimination or reduction of indigenous lands and slowness in their demarcation. Furthermore, this condition has caused families to lose their traditional habits^19^, particularly those concerning diet, and to consume industrialized products that are available due to external influences.

The anchoring of social representations of care in health care work reiterates the complexity of work involving indigenous health care. Moreover, it reveals the precariousness of working conditions, which itself indicates the great distance between the ideal health service model that the creation of the subsystem and the implementation of the NPHCIP are intended to achieve and the real world experienced in everyday work. This finding corroborates the results of other studies^7^ ^,^ ^13^ ^,^ ^20^ on the limited professional training of workers regarding the development of skills in intercultural contexts^18^.

One of the limitations of this study is the use of a qualitative method that does not permit generalization of the results. However, the use of a qualitative method allowed a deeper understanding of the process of formation of social representations of care for the Mbyá people in a specific context. This understanding emphasizes the need for changes in health care practices, particularly the need to expand the use of the concept of intermedicality by multidisciplinary teams with a focus on nurses, who are responsible for most of the health care work.

## Final Considerations

The study of social representations of care among IHMT workers expresses the tension between the care practices of traditional medicine and those of biomedicine despite the adoption of approaches for the promotion of intermedicality. A new basis for indigenous health care needs to be established by understanding the needs identified and promoting cooperation among individuals, communities, and health professionals in an intercultural context.
